# Recent Nitrogen Storage and Accumulation Rates in Mangrove Soils Exceed Historic Rates in the Urbanized San Juan Bay Estuary (Puerto Rico, United States)

**DOI:** 10.3389/ffgc.2021.765896

**Published:** 2021-11-12

**Authors:** Cathleen Wigand, Autumn J. Oczkowski, Benjamin L. Branoff, Meagan Eagle, Alana Hanson, Rose M. Martin, Stephen Balogh, Kenneth M. Miller, Evelyn Huertas, Joseph Loffredo, Elizabeth B. Watson

**Affiliations:** 1 Atlantic Coastal Environmental Sciences Division, United States Environmental Protection Agency (US EPA), Narragansett, RI, United States; 2 Gulf Ecosystem Measurement and Modeling, United States Environmental Protection Agency (US EPA), Gulf Breeze, FL, United States; 3 Woods Hole Coastal and Marine Science Center, United States Geological Survey, Woods Hole, MA, United States; 4 Oak Ridge Institute for Science and Education, Oak Ridge, TN, United States; 5 General Dynamics Information Technology, Alexandria, VA, United States; 6 Caribbean Environmental Protection Division, United States Environmental Protection Agency (US EPA), Guaynabo, PR, United States; 7 Department of Biodiversity, Earth & Environmental Science, The Academy of Natural Sciences, Drexel University, Philadelphia, PA, United States

**Keywords:** nitrogen storage, nitrogen accumulation, mangrove forest, wastewater, anthropogenic stressors, peri-urban mangrove, urbanization

## Abstract

Tropical mangrove forests have been described as “coastal kidneys,” promoting sediment deposition and filtering contaminants, including excess nutrients. Coastal areas throughout the world are experiencing increased human activities, resulting in altered geomorphology, hydrology, and nutrient inputs. To effectively manage and sustain coastal mangroves, it is important to understand nitrogen (N) storage and accumulation in systems where human activities are causing rapid changes in N inputs and cycling. We examined N storage and accumulation rates in recent (1970 – 2016) and historic (1930 – 1970) decades in the context of urbanization in the San Juan Bay Estuary (SJBE, Puerto Rico), using mangrove soil cores that were radiometrically dated. Local anthropogenic stressors can alter N storage rates in peri-urban mangrove systems either directly by increasing N soil fertility or indirectly by altering hydrology (e.g., dredging, filling, and canalization). Nitrogen accumulation rates were greater in recent decades than historic decades at Piñones Forest and Martin Peña East. Martin Peña East was characterized by high urbanization, and Piñones, by the least urbanization in the SJBE. The mangrove forest at Martin Peña East fringed a poorly drained canal and often received raw sewage inputs, with N accumulation rates ranging from 17.7 to 37.9 g ^−2^ y^−1^ in recent decades. The Piñones Forest was isolated and had low flushing, possibly exacerbated by river damming, with N accumulation rates ranging from 18.6 to 24.2 g ^−2^ y^−1^ in recent decades. Nearly all (96.3%) of the estuary-wide mangrove N (9.4 Mg ha^−1^) was stored in the soils with 7.1 Mg ha^−1^ sequestered during 1970–2017 (0–18 cm) and 2.3 Mg ha^−1^ during 1930–1970 (19–28 cm). Estuary-wide mangrove soil N accumulation rates were over twice as great in recent decades (0.18 ± 0.002 Mg ha^−1^y^−1^) than historically (0.08 ± 0.001 Mg ha^−1^y^−1^). Nitrogen accumulation rates in SJBE mangrove soils in recent times were twofold larger than the rate of human-consumed food N that is exported as wastewater (0.08 Mg ha^−1^ y^−1^), suggesting the potential for mangroves to sequester human-derived N. Conservation and effective management of mangrove forests and their surrounding watersheds in the Anthropocene are important for maintaining water quality in coastal communities throughout tropical regions.

## INTRODUCTION

Mangrove tidal forests have been referred to as the “coastal kidneys” of tropical and sub-tropical estuaries, because of their capacity to filter nutrients, baffle sediments, and provide water quality benefits (Duke, 2011). Worldwide and over the past century, mangrove systems have been under threat from land clearing and/or filling for agriculture, aquaculture, and urbanization purposes (Valiela et al., 2001; Duke et al., 2007; Duke, 2011). Peri-urban mangroves (i.e., adjacent to urban areas) are threatened by elevated contamination from sewage and domestic wastewater inputs (i.e., household water from laundry, dishwashing, and bathing), industrial wastes, storm overflow, and sediment runoff (Bosire et al., 2014; Lugo et al., 2014; Branoff, 2017, 2019; Pérez et al., 2018).

Human activities have greatly increased the flow of reactive nitrogen (N) from land to coastal marine ecosystems, often resulting in coastal eutrophication and causing adverse effects to the structure and function of wetland ecosystems (e.g., Howarth et al., 1996; Deegan et al., 2012; Reis et al., 2017). In a study comparing Puerto Rico rivers draining watersheds ranging from urbanized to more pristine and forested, McDowell et al. (2019) reported that urbanization had caused large increases in the concentration and flux of dissolved N and phosphorus (P) (2 - to 50-fold) in the urban river. In peri-urban mangrove systems, soil fertility can be elevated due to human activities and their associated waste products (e.g., sewage and industrial) (Pérez et al., 2018; Perez et al., 2020). Elevated rates of N and P accumulation were reported in peri-urban mangroves in southeastern Brazil (Sanders et al., 2014; Perez et al., 2020). It is not well understood how N is stored and cycled in peri-urban mangrove systems, although a recent meta-analysis suggests that leaf N content depends on surrounding land cover, with N enrichment in urban systems (Branoff, 2017). Sediment concentrations of heavy metals were also positively correlated with urban development, possibly having some effect on mangrove functions, including N cycling (Branoff, 2017).

The urbanized San Juan Bay Estuary (SJBE) has mangrove systems with varying hydrological alterations (e.g., dredged vs. clogged), levels of urbanization, sewage inputs, and landscape setting (Branoff, 2019; Oczkowski et al., 2020a). We previously found unexpected differences in N storage and cycling in the estuarine subtidal sediments associated with systems of varying degrees of urbanization in the SJBE (Oczkowski et al., 2020b). While subtidal sediments in the most urbanized area (the Caño Martín Peña) contained the most N, they also had the lowest N stable isotope values (Oczkowski et al., 2020a,b). This was surprising because urban runoff is often associated with elevated N isotope values. While the exact mechanisms are still being identified, we hypothesize that N fixation, which is an important source of N to mangroves, may be enhanced in these sediments by the ample supply of carbon (C) inputs from sewage and other urban sources (Oczkowski et al., 2020a,b). These studies also suggested that the San José Lagoon located in the innermost stretch of the SJBE may function as a settling basin and accumulate allochthonous and autochthonous inputs.

San Juan Bay Estuary mangrove forests had soil C burial rates ranging from 88 g ^−2^y^−1^ at the San José lagoon to 469 g ^−2^ y^−1^ at the Martin Peña East in recent decades (Wigand et al., 2021). Recent C burial rates were ~2.5 – 3 times greater than historic decades in mangrove forests at Piñones and Caño Martin Peña (Wigand et al., 2021). Differences in C sequestration rates among the SJBE mangrove systems were attributed to multiple factors: landscape setting, hydrology, vegetation, flooding regime, and anthropogenic inputs (e.g., runoff and sewage). Some changes in mangrove geomorphology and hydrology were associated with rapid urbanization in the SJBE watershed, including canalization and dredging among other disturbances (Lugo et al., 2014; Branoff, 2020a). In the present study, we examine patterns of N storage and accumulation in these same mangrove systems of the urbanized SJBE.

We tested for differences in N accumulation rates among SJBE mangrove sites of varying geomorphology, hydrology, nutrient fertility, and land development. We hypothesized that the mangrove stands in the most developed areas will have the highest sediment %N, N storage, and accumulation rates. However, the drivers of the N cycling and storage are complex and may be modulated by landscape setting, vegetation cover, varying soil characteristics (e.g., salinity, pH, and nutrient content), specific form of N inputs, and hydrology at the sites (Twilley and Day, 1999; Bowen et al., 2020; Perez et al., 2020). Therefore, to assess N dynamics we also reported the dissolved nutrients, salinity, and pH in the porewater, and the %N, molar *C/N* ratios, and N and sulfur (S) stable isotope ratios in the sediments at each site. We used published sediment accretion rates and bulk densities from the same mangrove sites to report on N storage and accumulation rates in recent (1970 – 2016) and historic (1930 – 1970) decades and estimated watershed-wide N storage and accumulation rates in the SJBE.

The magnitude of N accumulation at both the mangrove site and SJBE watershed-wide scale were compared with population density, food inputs, and associated human wastes to assess the potential socio-ecological links between mangrove N accumulation rates and human-sourced N. The SJBE watershed (area: 41,572 ha) currently has the densest human population on the island, ~1,850 people k^−2^, primarily located in the urban municipalities (i.e., San Juan, Carolina, and Bayamón) associated with the city of San Juan, Puerto Rico (U.S. Census Bureau, 2017). In addition to degree of urbanization, landscape setting, and flushing, we discuss different biogeochemical factors in the mangrove soil and porewater that may influence N storage and accumulation rates. An understanding of N-cycling in peri-urban mangrove systems, often subject to anthropogenic stressors, will support effective land management efforts to sustain mangrove systems and the services they provide.

## MATERIALS AND METHODS

### Site Description

**FIGURE 1 |:**
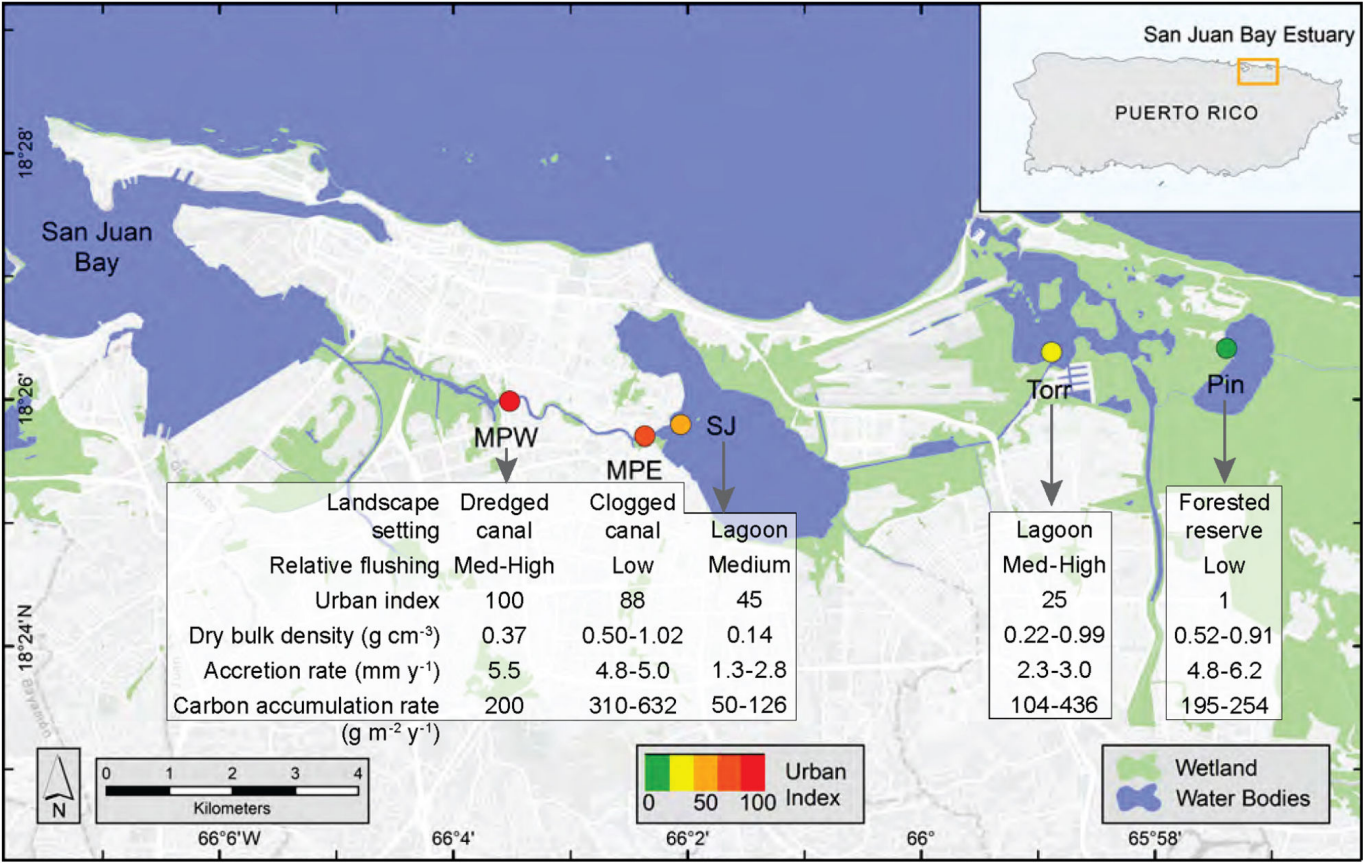
Map of San Juan Bay Estuary with study sites, urban index (colored circles), landscape setting, relative tidal flushing, and mangrove soil characteristics. The Urban Index was scored on a scale from 1 to 100 and based on surrounding coverage of mangrove wetland, non-mangrove vegetation, open water, urban land, population density, and road density, which were determined in a 500 m buffer from the approximate center of the study site. National Wetland Inventory data were used to depict wetlands, which included estuarine and marine, freshwater emergent, and freshwater forested/shrub wetlands (U.S. Fish and Wildlife Service, 2018). Tidal flushing and mangrove soil characteristics were based on previously published data (Branoff, 2020a; Eagle et al., 2021; Wigand et al., 2021); see Table 1 for site abbreviations.

The five SJBE mangrove study sites were located along a previously established urbanization gradient spanning the highly developed western end to the less developed eastern end, which contains the largest remaining mangrove forest on the island (Figure 1; Oczkowski et al., 2020a). The relative tidal flushing, landscape setting, soil characteristics, and degree of urbanization among the different mangrove forests had been previously reported and was based on water level observations, assessments of the landscape geomorphology, and detailed analyses of hydrology, land use, and population density (Branoff, 2020a; Martin et al., 2020). The subtidal sediments had variable grain size, but consistently low (1 – 17%) carbonate content (Oczkowski et al., 2020a).

The urban index, expressing the degree of urbanization (1 being least and 100 most urbanized), was derived from assessments of impervious surfaces, population density, vegetation coverage, and road density (Branoff, 2020a). In the present study the urban indices were determined for the 500 m buffer from the approximate center of each mangrove study site and ranged from 1 at Piñones Forest to 100 at Martin Peña West (Figure 1; Branoff, 2020a). Some mangrove soil characteristics including dry bulk density (range: 0.14–1.02 g cm^−3^), sediment accretion rates (range: 1.3–6.2 mm y^−1^), and carbon accumulation rates (50–632 g ^−2^ y^−1^) have been earlier reported for these same SJBE mangrove sites (Figure 1; Eagle et al., 2021; Wigand et al., 2021).

### Core Collections and Analyses

The present study of mangrove N storage and accumulation was part of parallel investigations of greenhouse gas emissions and carbon sequestration at the same sites (Martin et al., 2020; Wigand et al., 2021). The raw radiometric data (^137^Cs, ^210^Pb, and ^226^Ra) and associated C, N mangrove core data from the earlier carbon sequestration study are available in a U.S. Geological Survey (USGS) data release (Eagle et al., 2021). We used the same 9 cores as previously described in the carbon sequestration study to estimate N storage and accumulation rates at the same mangrove sites. Mangrove sediment cores, two from each site about 1 m apart, were collected in March 2016. Study resources allowed only duplicate cores from each site for the radiometric and isotopic analyses to obtain a historic record of N fertility and stable isotopic signatures. Cores were collected in the low intertidal zone and were selected to be representative of the mangrove study site. The mangrove sites sampled included the western Martin Peña (MPW), eastern Martin Peña (MPE), San José Lagoon (SJ), La Torrecilla Lagoon (Torr), and Piñones Lagoon (Pin). Cores were collected with a Macaulay peat sampler (Jowsey, 1966) to a maximum depth of 50 cm depending upon ability to penetrate coarse mangrove rhizomes. Core depths ranged from 37 to 50 cm. One core collected from MPW was damaged during transport and was not radiometrically dated. Most cores were sliced in 1-cm segments in the surface (0–3 cm) and then every 2 cm to the bottom of the core unless otherwise indicated. Two cores (one each from MPE and Torr) were sliced every 2 cm. Sediment subsamples from the nine cores were used to measure stable N isotope ratios, measure percent C and N, and estimate N storage and N accumulation rates. An additional core from each of the 5 mangrove sites was collected on a separate visit (June 2016) and used to examine sulfur (S) stable isotope ratios with depth. These additional cores were collected in a similar fashion as described above with a peat sampler and sliced in 1-cm increments in the surface (0–3 cm) and then every 2 cm to the bottom of the core.

### Processing for Percent C, N, and Stable Isotopes

Sediment subsamples were dried at 60°C for at least 48 h then ground to a fine powder using a mortar and pestle before stable isotope (N, S) analyses. Subsamples to be used for measurements of %C and δ^13^C were fumigated prior to analyses with 12 M HCl to remove carbonates, following Harris et al.(2001). Subsamples for %N, %S, and N, S isotope analyses were not fumigated. The N isotope compositions were determined using an Elementar Vario Micro elemental analyzer connected to a continuous flow Isoprime 100 isotope ratio mass spectrometer (IRMS) (Elementar Americas, Mt. Laurel, NJ, United States). Replicate analyses of isotopic standard reference materials USGS 40 (δ^13^C = −26.39 ‰; δ^15^N = −4.52‰) and USGS 41 (δ^13^C = 37.63h; δ^15^N = 47.57 ‰) were used to normalize isotopic values of working standards (blue mussel homogenate) to the air (δ15N) and Vienna Pee Dee Belemnite (δ^13^C) scales (Paul et al., 2007). Analysis of carbon storage and dominant carbon sources to the SJBE mangrove soils determined from these same cores were reported earlier (Eagle et al., 2021; Wigand et al., 2021).

Sulfur stable isotopes (δ^34^S) were measured in sediments at the Center for Stable Isotope Biogeochemistry at the University of California at Berkeley where analyses followed the SO_2_ EA-combustion-IRMS method. The profiles of δ^34^S with depth for each site were modeled with quadratic and linear model types to determine best fit. Subsequently, comparisons of the site-specific model terms for profiles of similar model types across sites were performed. Comparisons were based on calculating confidence bounds for each model term (intercept, slope and quadratic term where applicable) and evaluating whether those bounds overlap for the different sites. Individual site confidence bounds were based on achieving an overall 95% confidence level (such that the probability of incorrectly calling any two sites different across all relevant comparisons would be held to 5%).

Stable isotope values are expressed in δ notation following the formula δ*X* (‰) = [(Rsample/Rstandard) − 1] × 10^3^, where *X* is the less common isotope and *R* is ratio of the less common to more common isotope [^15^N/^14^N or ^34^S/^32^S]. During analyses, working standards were analyzed after every 24 samples to monitor instrument performance and check data normalization. The precision of the laboratory standards was better than ± 0.3‰ for δ^15^N, and better than ±0.2 ‰ for δ^34^S values. The %C and %N were calculated by comparing the peak area of the unknown sample to a standard curve of peak area versus the C or N content of a known standard.

### Porewater Nutrients and Soil Salinity, pH, and Temperature

We collected sediment porewater using a push point sampler^1^ (East Tawas, MI, United States) at depths of 15 cm in the wet season (June 2016), but sometimes deeper (20–30 cm) in the dry season (February 2017). Filtered (GF/F) porewater samples were acidified and frozen for later analyses of ammonium (NH_4_+), nitrate and nitrite, and phosphate (PO_4_^3−^) using an Astoria-Pacific Astoria 2 continuous-flow analyzer (Astoria-Pacific, Clackamas, OR, United States), following standard methods (US EPA methods 353.2 and 350.1). Samples were calibrated against a five-point standard curve, with check standards run every 15 samples and Milli-Q blanks every 10 samples. Nitrate and nitrite values were very low and sometimes non detectable and were summed with NH_4_+ to report dissolved inorganic nitrogen (DIN). A two-way ANOVA was used to evaluate the site and seasonal effects on the porewater nutrients (NH_4_+, DIN, and PO_4_^3−^) among the mangrove sites; with the Bonferroni pairwise comparison approach subsequently employed to identify specific differences among sites and seasons. Results were natural log-transformed prior to performing the analyses to ensure that the model’s statistical assumptions of the data following a normal distribution were met (model residuals yielded *p* > 0.05 for all nutrients, based on the Shapiro–Wilk test for normality). These analyses were performed on data with nitrate and nitrite non-detects handled in two ways (set to 0 and the detection limit), and it was confirmed that the non-detect approach had no impact on the statistical results. Final results are presented with 0 used for non-detects, because this gives a more conservative estimate of the variability in the data.

Surface soil temperature was similar among the SJBE mangrove sites: 25°C in the dry season (February 2017) and 27–29°C in the wet season (June 2016) (Martin et al., 2020). Porewater salinity and soil pH were measured in an earlier report from the same SJBE mangrove sites (Martin et al., 2020; Table 1).

### Site and Estuary-Wide N Storage and Accretion Rates

Age-depth models, dry bulk density, and sediment accretion rates (SAR) were previously described for each core and mangrove site (Figure 1; Eagle et al., 2021; Wigand et al., 2021). These age-depth models reported apparent ages and accretion rates since they incorporated any post-depositional processes, including diagenesis and compaction, that may occur. The sediment accretion rates were used to determine the N accumulation rates (SAR*dry bulk density*%N) with depth for each core in the present study. N storage was estimated as the product of bulk density, depth and fraction N. The N storage and accumulation rates as well as N stable isotopes, within and among sites, were compared for two decadal periods of interest, recent decades (1970–2016) and historic ones (1930–1970). The defined historic period (1930s–1970s) coincides with a period of mangrove recovery following over a century of intense agricultural activity, including the conversion of lands to sugar cane fields and pastures in the 1800s (Martinuzzi et al., 2009). The recent time period (1970s to present-day) represented a period characterized by rapid urbanization with watershed land development and increases in population density, but also adoption in 1972 of legal protections for all mangroves on the island (Martinuzzi et al., 2009).

The SJBE watershed-wide mangrove N storage and accumulation rates were estimated by interpolating above and belowground measurements onto a 2 m × 2 m grid of mangrove areas as represented by the “estuarine forested wetlands” class in the NOAA C-CAP dataset (Office for Coastal Management, 2017). Belowground soil N measurements were taken from the present study while aboveground measurements were estimated from SJBE biomass measurements taken from Branoff and Martinuzzi (2020) for aboveground stocks and Branoff (2020b) for aboveground accumulation. Aboveground N content within a stand was taken as a weighted mean between the proportion of biomass represented by a given species and the average aboveground N content of each species in the stand. Direct measures of N content in SJBE aboveground biomass were not available, so South Florida N values reported by Romero et al. (2005) for the three mangrove species (*Avicennia germinans* = 0.26%, *Laguncularia racemosa* = 0.157%, and *Rhizophora mangle* = 0.205%) and Luquillo Experimental Forest (Puerto Rico) values reported by Scatena et al.(1993) for the non-mangrove species (0.31%) were used in our estimates. Values throughout the estuary were then interpolated between the known values at each site through an inverse distance weighting methodology (Shepard, 1968) via the *idw* function of the phylin package in R (Tarroso et al., 2015). This interpolation incorporates all known values into the estimate but gives higher weight to the geographically closest values. Values were randomly drawn from right skewed normal distributions that mimicked those of the cores at each site, using the same means and standard deviations from the core measurements via the rsnorm function of the fGarch package in R (Wuertz et al., 2020). All grid values of each N component (aboveground, recent soils, historic soils) were then summed to give SJBE watershed-wide estimates of total N stocks and accumulation rates across the estuary. This was repeated for 100 iterations and final reported values represent the mean and standard errors of the sums from each iteration.

### Bootstrap Methods for Mangrove Site and Time Period Comparisons

Mean SAR, dry bulk density, % N, and δ^15^N were calculated within two identified time periods: recent decades, approximately the 1970s to present-day (i.e., 2016, date of collection) and historic decades (approximately the 1930s to 1970s). Time periods were earlier determined from the simulated means in the age-depth models generated by radiometric dating (Eagle et al., 2021; Wigand et al., 2021). We used a bootstrap approach to generate means and 95% confidence bounds for each parameter within sites, cores, and specific time periods. The bootstrap analysis generated 1,000 sets of randomly selected data from the reported values for the specific time period in a given core (Efron and Tibshirani, 1993). The mean of those 1,000 values was used as the overall mean estimate for the parameter, and the 2.5th and 97.5th percentiles of those 1,000 values were the upper and lower confidence bounds.

Using the previous estimates of SAR and dry bulk density for the SJBE mangrove cores (Eagle et al., 2021; Wigand et al., 2021) and %N from the present study, we calculated N accumulation rates and N storage for each time period. To propagate the uncertainty associated with each individual measurement included in the calculation of these parameters, the bootstrap generated 1,000 sets of randomly selected pairings of the variables going into the calculation. For example, the N accumulation rate calculation was based on randomly selecting a SAR, dry bulk density and a %N value from the same core in a specific time period, but not necessarily with each coming from the same depth. By not forcing the SAR, bulk density and %N to come from the same depth, any additional variability resulting from depth/temporal impacts varying among the three parameters would be included in the bootstrap evaluation. The means and 2.5th and 97.5th percentiles of the 1,000 sets of calculated values would be the overall mean and confidence bounds, as with the other parameters.

For examination of spatial comparisons among mangrove sites, the 1,000 bootstrap estimates from both core replicates from a specific site were combined to calculate site-specific means and bounds. The mean of those 2,000 values was used as the overall mean estimate for the parameter, and the 2.5th and 97.5th percentiles of those 2,000 values were the upper and lower confidence bounds. The site-specific bounds generated by combining the bootstrap estimates tended to be wider than the individual core-specific bounds when the variability among the two cores was large. However, comparisons made based on these site bounds will enable more meaningful evaluations of spatial differences.

For temporal comparisons, because core variability can mask changes over time within individual core locations, we examined whether significant differences existed between recent vs. historic time period means using the confidence bounds generated based on each of the 1,000 core-specific bootstrap estimates. For both the spatial and temporal comparisons, significant differences were evaluated based on whether bootstrap bounds (2.5th and 97.5th percentiles) overlapped. For N storage, comparisons between recent (1970–2016) and historic time periods (1930–1970), values were normalized to account for the difference in range between time periods (46 vs. 40 years).

### Site and San Juan Bay Estuary Watershed N Inputs in Food

Material and energy flow analysis was used to estimate the magnitude of food N inputs attributed to people in the 500 m buffers adjacent to the mangrove sites and the entire SJBE watershed (Kennedy et al., 2015). We first estimated N input per person from food consumption. Subsequently, we quantified food-waste export flows to local groundwater and nearby coastal waters and/or the ocean (Supplementary Table 1). The estimate included total N input from food and N exported as food waste in the municipal solid waste stream, recycled in compost, and wastewater. Estimates were based on population within a 500m buffer associated with the mangrove sites and for the entire SJBE watershed (population: 769,000). To estimate N inputs from food, we multiplied the population in the mangrove buffer or in the entire SJBE watershed by food demand per person in kilograms (U.S. Department of Agriculture Economic Research Service, 2015a), then multiplied by the average N content of food (2.45%, U.S. Department of Agriculture Economic Research Service, 2015b; Supplementary Table 1). The export of N from food intake was allocated among municipal solid waste (38%, U.S. Department of Agriculture Economic Research Service, 2015a, 2016), and wastewater that was either transported to treatment plants, and eventually exported to the ocean (44%), or treated in backyard septic systems (18%) (Meléndez-Ackerman et al., 2016). In some neighborhoods, raw sewage flowed directly into the stormwater system or into the groundwater (Bunch et al., 2000; Oczkowski et al., 2020a). We recognize that there is an N stock exchange due to growing humans, but assume it is negligible compared to the annual wastewater flows. In the input-export N flows in the present study we assumed the N stock change in humans to be zero. To allow comparison between sites and between natural stocks and flows of N, we converted flows into N Mg ha^−1^ y^−1^ by dividing by the area of interest (500 m buffer area or SJBE watershed area: 41,572 ha).

## RESULTS

### Mangrove Porewater

In the present study, we observed significantly greater NH_4_+ and DIN concentrations in porewater at Piñones compared to the other mangrove sites, and in an earlier study, we reported lower pH and higher salinity at Piñones (Table 1; Martin et al., 2020). There were significant seasonal effects (Two-way ANOVA, season × site interaction *P* < 0.0001, followed by pairwise comparisons *P* < 0.0025) on porewater nutrients among some of the sites (PO_4_^3−^: Martin Peña West and Piñones, Dry > Wet; NH_4_+: Martin Peña East Dry > Wet; DIN: Martin Peña East Dry > Wet, Piñones Wet > Dry). In the wet season, porewater DIN and NH_4_+ concentrations were significantly greater in Piñones than all other sites, and in the dry season, comparisons among sites showed Piñones > Martin Peña East > Martin Peña West, San José Lagoon, La Torrecilla (Bonferroni pairwise differences, *P* < 0.01). Highest porewater phosphate was measured at Martin Peña East in the wet and dry season. Porewater PO_4_^3−^ comparisons among sites resulted in significant differences: Martin Peña East > Piñones > Martin Peña West, San José Lagoon, La Torrecilla in the dry season and Martin Peña East > all other sites, La Torrecilla > Martin Peña West in the wet season (Bonferroni approach, *P* < 0.01).

The porewater *N:P* molar ratios suggested N limitation in wet and dry seasons in the Martin Peña East (*N:P* ratios: 0.5:1, Wet; 1:1, Dry) and to a lesser extent, N limitation at La Torrecilla (*N:P* ratio 3:1, Wet; 2.5:1, Dry) and San José lagoon (*N:P* ratio 10:1, Wet; 6:1, Dry) (Table 1). While there was apparent N limitation at the clogged, Martin Peña East, the porewater *N:P* ratio at the dredged, western end of the canal, Martin Peña West (*N:P* ratio 15:1 Wet; 11:1, Dry) approached reported mangrove porewater *N:P* ratios (~19:1, Alongi, 1996). In contrast, at Piñones Forest the porewater *N:P* molar ratio of 271:1 in the wet season suggested severe P limitation. The *N:P* ratio of 14:1 in the dry season at Piñones approached the previously reported porewater *N:P* ratio of low intertidal mangrove forests (Alongi, 1996).

### Mangrove Soil %N and Molar *C:N* Ratios

During historic decades (1930 – 1970s) the mangrove soil from the San José Lagoon site had significantly greater %C (31.2%) and %N (0.97%) and the highest *C:N* ratios (37.6) compared to all other sites (Supplementary Table 2). The historic molar *C:N* ratios (13.1) of the Piñones mangrove soils were lower than all the other sites. The mangrove soil %N in recent decades was again greatest at San José lagoon (1.24 %N) compared to all other sites; La Torrecilla (0.76% N) and Martin Peña East (0.74% N) were of similar magnitude; and Martin Peña West (0.49% N) was significantly lower than all other sites (Supplementary Table 2). The mangrove soil molar *C:N* ratio in recent decades was lowest at Piñones (12:1) followed by Martin Peña East (20:1), and the *C:N* ratios (ranging from 23.4 to 27.8) were of similar magnitude at Martin Peña West, San José Lagoon, and La Torrecilla Lagoon. All mangrove soils sampled showed recent sediment %N greater than historic %N except at Martin Peña West where there was no difference between time periods.

### N Storage at Mangrove Sites

**FIGURE 2 |:**
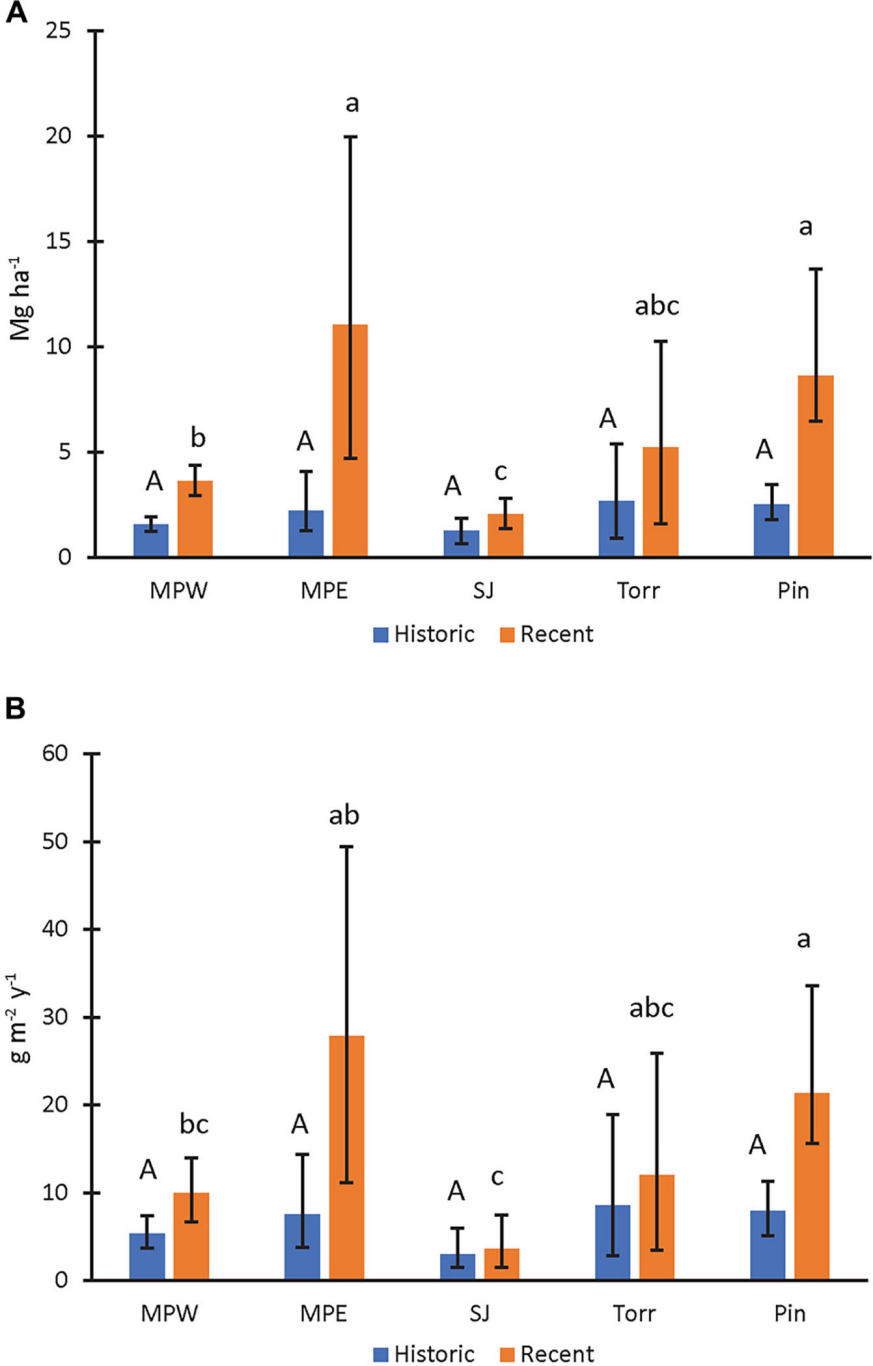
Mangrove soil nitrogen storage **(A)** and accumulation rates **(B)** in recent (1970–2016) and historic (1930–1970) decades. Upper-case letters were used to describe mangrove site comparisons within historic decades and lower-case letters to describe site comparisons within recent decades. Mangrove sites that do not share letters within a specific time period had significantly different (*P* < 0.05) values. Statistical site differences within time periods were based on whether bootstrapped bounds (i.e., 2.5th and 97.5th percentiles) overlapped. Parameter means, lower, and upper confidence bounds (bars) were generated on bootstrap runs (1,000 bootstrap values per core; combined for a total of 2,000 values for sites with two replicates). Sites listed from high to low urbanization index (see Table 1 for site abbreviations).

Although the San José cores had significantly greater soil %N than all the other sites in historic and recent decades, the dry bulk densities and sediment accretion rates were relatively low compared to the other sites (Figure 1), which resulted in generally low N storage and accumulation rates (Figures 2A,B). The N storage ranged from 1.27 to 2.69 Mg ha^−1^ among the mangrove forest sites in historic decades, and there was no significant difference among sites (Figure 2A).

The N stocks ranged from 2.06 to 11.07 Mg ha^−1^ among the SJBE mangrove sites in recent decades (Figure 2A). N storage in the mangrove soils of San José Lagoon were significantly lower than the other sites in recent decades (Figure 2A). In addition, Martin Peña East and Piñones mangrove soil N stocks were significantly greater than Martin Peña West. The N storage of La Torrecilla Lagoon was similar in magnitude to Martin Peña East and Piñones. Recent N storage was significantly greater than historic storage for all the cores, except those collected from the San José Lagoon and La Torrecilla Lagoon.

### N Accumulation Rates at Mangrove Sites

The historic N accumulation rates in the mangrove forest soils ranged from 2.98 to 8.59 g ^−2^ y^−1^, and there was no difference in accumulation rates among sites in historic decades (Figure 2B). The recent N accumulation rates ranged from 3.67 to 27.89 g ^−2^ y^−1^ among the mangrove sites, with greatest accumulation rates at Piñones Forest and the rates of Martin Peña East and La Torrecilla Lagoon of similar magnitude (Figure 2B). San José Lagoon had the lowest recent soil N accumulation rates, which were 63–87% lower than the N accumulation at three other sites: Martin Peña West, Piñones Forest, and Martin Peña East. The recent N accumulation rates were 170–270% significantly greater than historical rates at Piñones Forest and Martin Peña East, respectively. There was no significant temporal change in N accumulation rates at the other sites.

### δ^15^N Profiles and Historic vs. Recent Trends

**FIGURE 3 |:**
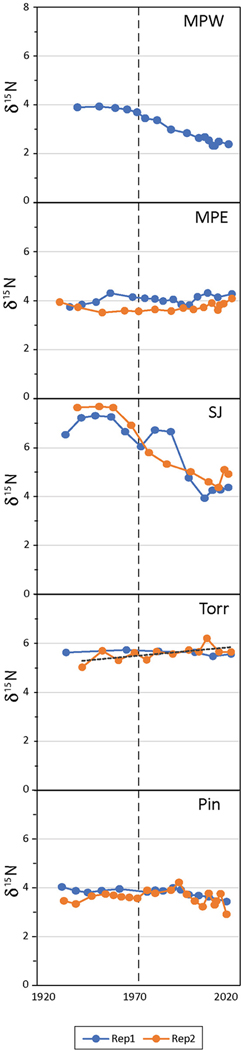
Mangrove soil bi-plot of sediment δ^15^N vs. time in radiometrically-dated mangrove cores (see Table 1 for site abbreviations; all sites had 2 core replicates except MPW).

Prior to the 1970s the mangrove soils of the San José Lagoon had significantly higher mean δ^15^N (~7‰) among all mangrove forest study sites in the SJBE, but in more recent decades the mean δ^15^N was lower ~5‰ and similar in magnitude to La Torrecilla Lagoon (Figure 3 and Supplementary Table 2). In historic decades the mean δ^15^N ranged from 3.8‰ at Piñones to 7.2‰ at San José Lagoon, while in recent decades the mean δ^15^N ranged from 2.8‰ at Martin Peña West to 5.6‰ at La Torrecilla Lagoon (Figure 3 and Supplementary Table 2). In historic decades Martin Peña West (3.9‰), Martin Peña East (3.9‰), and Piñones (3.8‰) were significantly lower than La Torrecilla Lagoon (5.6‰) and San José Lagoon (7.2‰). In recent decades Martin Peña West (2.8‰) was significantly lower than all the other mangrove sites, Martin Peña East (3.9‰) and Piñones (3.7‰) were intermediate, and San José Lagoon (5.1‰) and La Torrecilla Lagoon (5.6‰) had the highest mean δ^15^N (Supplementary Table 2). San José Lagoon and Martin Peña West cores had greater mean δ^15^N in historic than recent decades, while cores from all other mangrove sites showed no difference in mean δ^15^N between time periods.

### δ^34^S Profile With Depth

**FIGURE 4 |:**
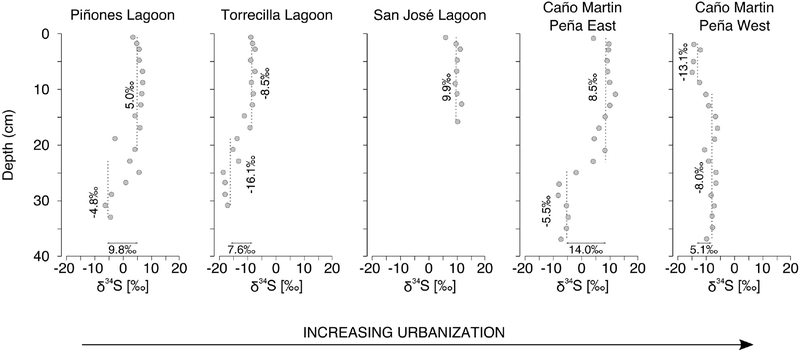
Mangrove soil depth profiles of δ^34^S at sites along an urbanization gradient in the San Juan Bay Estuary.

Piñones Forest, La Torrecilla Lagoon, and Martin Peña East, all had δ^34^S values that increased toward the core surface. In contrast, δ^34^S decreased toward the core surface at the Martin Peña West site (Figure 4). A curvilinear relationship (quadratic model) of δ^34^S with depth was the best fit for all sites, except for the linear profile of San José Lagoon, which was the shortest core. In among site comparisons of the curvilinear relationships, δ^34^S intercepts among sites were significantly different (Martin Peña East, Piñones > La Torrecilla Lagoon > Martin Peña West; Figure 4) indicating differences in surface-depth concentrations. However, there was no difference in the other (depth) quadratic terms indicating no clear systematic differences in the rate of change with increasing depth. We observed no pattern of δ^34^S isotope profiles with the urbanization gradient; however, the sites with the greatest tidal flushing (Martin Peña West, La Torrecilla Lagoon) had the lowest δ^34^S, and the sites with the least flushing (Piñones, Martin Peña East) had the highest values, suggesting that the degree of tidal flushing contributes to sulfur isotopic variation.

### Estuary-Wide N Storage and Accretion

**FIGURE 5 |:**
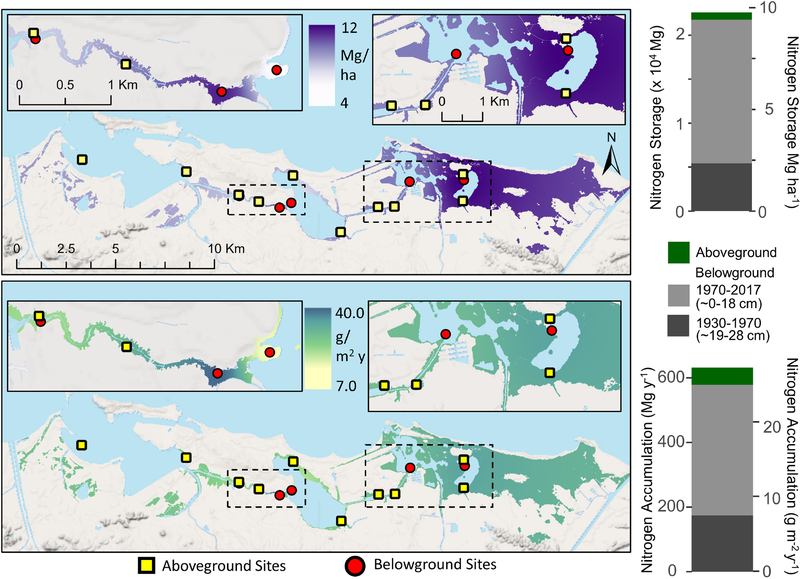
Interpolated mangrove forest nitrogen storage **(top)** and accumulation rates **(bottom)** for both aboveground biomass and belowground soil (~28 cm depth) components in the San Juan Bay Estuary. Map values represent distance weighted interpolations between known measurements at site locations. Bar graphs represent sums of all mangrove areas across the SJBE watershed. Aboveground mangrove sites (Branoff and Martinuzzi, 2020) are denoted by squares and belowground sites (this study) by circles.

The total SJBE watershed-wide N storage estimate in aboveground mangrove was 830.7 ± 18.9 Mg or 0.36 ± 0.008 Mg ha^−1^ (Table 2). Aboveground N accumulation rates ranged from 1.7 to 3.0 g ^−2^ y^−1^, reflecting differences in mangrove species composition and growth rates between the sites (Figure 5). The watershed-wide total mean aboveground N accumulation rate was 52.7 Mg y^−1^, with an areal mean of 0.02 Mg ha^−1^y^−1^ or 2.0 g ^−2^ y^−1^ (Figure 5 and Table 2).

Most (96.3%) of the mangrove N (9.4 Mg ha^−1^) was stored in the soils with 7.1 Mg ha^−1^ sequestered in the most recent time period (1970–2017, 0–18 cm) and 2.3 Mg ha^−1^ in the historic period (1930–1970, 19–28 cm) (Table 2 and Figure 5). The estuary-wide aboveground N storage represents only 3.8% of the total ecosystem N storage up to 28 cm of soil depth, exemplifying the importance of belowground stocks in mangrove N pools. As for the watershed-wide soil N accumulation, we estimated rates in the recent time period (0.18 ± 0.002 Mg ha^−1^y^−1^ or 18 g ^−2^ y^−1^) more than twice as high as the historic period (0.08 ± 0.001 Mg ha^−1^y^−1^ or 8 g ^−2^ y^−1^) (Table 2).

### Food N Inputs and Associated Wastewater and Solid Wastes

Nearly 5,400 Mg N yr^−1^ enter the SJBE watershed (area: 41,572 ha) from food inputs into the system (Table 3 and Supplementary Table 1). Approximately four-fifths of the food inputs are imported to Puerto Rico, and one-fifth of the inputs are provided by on-island farms (Supplementary Table 1). When expressed as an areal rate the intensity of food N inputs ranged 100-fold from 0.004 Mg ha^−1^ y^−1^ in the least populated and relatively pristine Piñones to 0.384 Mg ha^−1^ y^−1^ in the densely populated, peri-urban Martin Peña East (Table 3 and Supplementary Table 1). Average N loading either directly or indirectly from food was 0.13 Mg ha^−1^ y^−1^ across the SJBE watershed. Approximately 1,924 Mg N is deposited annually in area landfills from food waste, and an additional 970 Mg N enters the groundwater through wastewater treated with septic systems across the SJBE watershed (Table 3). The remainder flows to wastewater treatment plants (2,371 Mg N), where only primary treatment takes place, resulting in no N or P removal, before being transported offshore and into coastal waters. The rate of SJBE watershed food N exported in wastewater (i.e., to coastal waters plus groundwater) was 0.08 Mg ha^−1^ y^−1^.

In a *post hoc* check of the assumption that N stock exchange due to growing humans was negligible, we assumed children (~20% of the SJBE watershed population) gained 2.5 kg per year, and the relative N portion of that was 2% (1.8 kg N/90.7 kg average mass). The 20% of the watershed population that were below age 18 each added 0.05 kg N to the stock of humans for a total of 7,690 kg or 7.7 Mg. This represented less than 0.5% of total annual wastewater flows.

## DISCUSSION

Human wastewater and other nutrient inputs can enhance the soil fertility and ultimately affect N storage in peri-urban mangroves. In addition, hydrological alterations and land development as well as natural drivers such as varying soil characteristics, salinity, and tidal regime among mangrove systems in the SJBE can affect N storage and accumulation rates. In our study, we measured some of the highest N storage (11.1 Mg ha^−1^) and accumulation rates (27.9 g ^−2^ y^−1^) in the highly urbanized and altered Martin Peña East in recent decades. Mangrove forests at Martin Peña East fringed a poorly drained canal and often received raw sewage, domestic wastewater, and stormwater overflow from the surrounding neighborhoods (Bunch et al., 2000; Oczkowski et al., 2020a). High N storage and accumulation rates were also measured in the least developed Piñones Forest (8.6 Mg ha^−1^, 21.2 g ^−2^ y^−1^, respectively) and the La Torrecilla Lagoon (5.2 Mg ha^−1^, 12.0 g ^−2^ y^−1^, respectively) with a dredged inlet and modified shoreline. Within site variability at La Torrecilla Lagoon was high with one core having significantly lower soil bulk density, sediment accretion rates, and N accumulation rates, suggesting high spatial variability associated with the dredging and shoreline modifications at that site.

**FIGURE 6 |:**
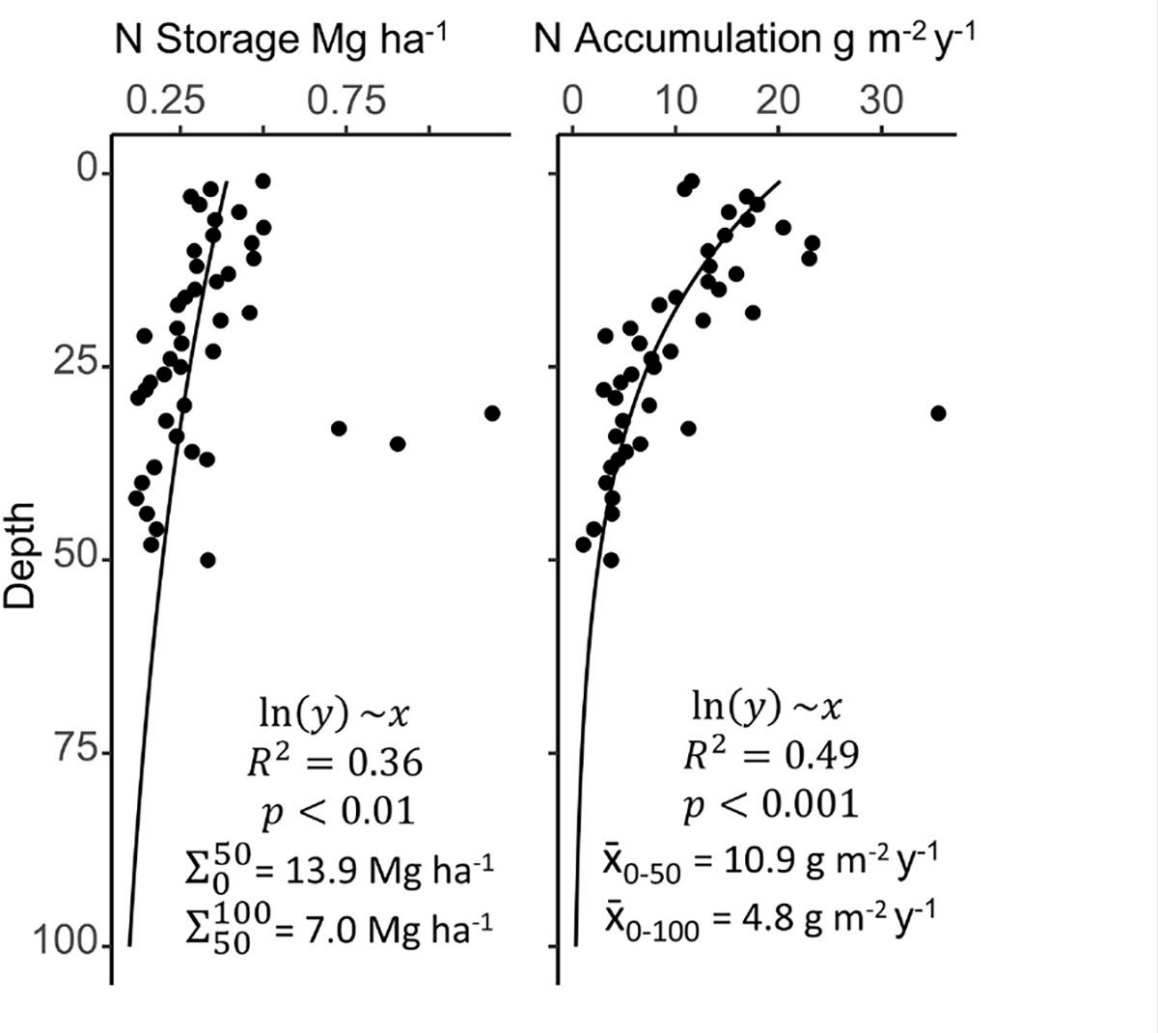
Mean depth profiles for nitrogen (N) storage and accumulation across all sites, with the deepest layers approximated by extrapolation of a log transformed linear model. Filled circles are combined mangrove soil data from nine San Juan Bay Estuary cores. Site-specific depth profiles (not shown) varied greatly and may not necessarily follow the estuary-wide generalizations.

While variable across the estuary, N storage in the SJBE falls within the range observed in other mangrove forests. The few published studies that provide direct measurement of N storage in mangrove soils suggest a range of 2.7–56 Mg ha^−1^, although the depths (i.e., 20–356 cm) and mangrove types corresponding to these N stocks vary widely (Fujimoto et al., 1999; Alongi et al., 2003; Khan et al., 2007; Bulmer et al., 2016). In the SJBE mangrove soils, N storage in recent decades ranged from 2.1 to 11.1 Mg ha^−1^ among sites, similar in magnitude to mangroves: in Rookery Bay (FL) ranging from 2.0 to 4.8 Mg ha^−1^, in southern Thailand ranging from 6.1 to 12.9 Mg ha^−1^, and in New Zealand averaging 14.6 ± 1.7 Mg ha^−1^ (Steyer, 1988; Alongi et al., 2004; Bulmer et al., 2016). The SJBE watershed-wide estimate of 9.4 Mg ha^−1^ fell at the lower end of the published range, but just represented 28 cm depth. By extending the trend in N storage to 50 and 100 cm depths for comparison, our SJBE estuary-wide estimates were 13.9 and 20.9 Mg ha^−1^, respectively (Figure 6). The estimate extrapolated to 100 cm depth aligns closely with another indirect estimate for global mangrove N storage of 20 Mg ha^−1^, although it is not clear what depth range this represents and the authors state this was an unmeasured estimate based on *C:N* ratios (Reis et al., 2017). Still, the 100 cm extrapolation values in our study ranged from 8.1 Mg ha^−1^ at San Jose to 28.6 Mg ha^−1^ at Martin Peña East, exemplifying the high spatial variability in N storage of mangrove soils within the same estuarine system.

When considering storage over the past 80 years, the rate of N accumulation in the SJBE was exceptionally high at the peri-urban Martin Peña East site and the most isolated and least urbanized Piñones Forest site. Mean N accumulation rates in the SJBE ranged from 3.0 to 8.6 g ^−2^ y^−1^ in historic decades and 3.7–27.9 g ^−2^ y^−1^ in recent decades. The N accumulation rates in recent decades at Piñones (21.3 g ^−2^ y^−1^) and Martin Peña East (27.9 g ^−2^ y^−1^) were elevated and ~2.7–3.7 times greater than historic decades. Nitrogen accumulation rates among riverine and basin forests in the Gulf of Mexico averaged 5.5 g ^−2^ y^−1^ (Lynch, 1989; Twilley and Day, 1999). The N accumulation rates in recent decades at Martin Peña East and Piñones were ~4 – 5 times greater than the average N accumulation rates among riverine and basin mangroves in the Gulf of Mexico. However, N accumulation rates in the highly impacted and eutrophic Cubatao Forest and Septiba Bay mangrove systems in southeastern Brazil were as high as 31 and 90 g ^−2^ y^−1^, respectively; likely because of high urban sewage discharge since the 1900s (Sanders et al., 2014; Perez et al., 2020). The Martin Peña East accumulation rates were similar in magnitude to the Cubatao Forest (Sanders et al., 2014). However, the Septiba Bay N accumulation rates were ~3 times greater than the Martin Peña East rates and ~5 times greater than the SJBE watershed-wide N accumulation rate (18 g ^−2^ y^−1^) in recent decades, suggesting that on the scale of the entire estuary, the SJBE may not be as impacted as other human-modified mangrove systems. When extrapolated down to 50 and 100 cm, SJBE watershed-wide belowground N accumulation rates averaged even lower to 10.9 and 4.8 g ^−2^ y^−1^, respectively; demonstrating the importance of recent N dynamics, likely anthropogenically driven, to the soil N pool (Figure 6).

We observed different N storage and accumulation rates at the western and eastern ends of the highly developed Caño Martin Peña. All SJBE mangrove sites exist against a backdrop of rising sea levels, with estimated rates for Puerto Rico of ~2 mm/year (NOAA station #9755371). Biogeomorphic feedbacks between rising water levels and mangrove organic matter production and decomposition results in an accommodation space within which organic matter preservation is enhanced (Gonneea et al., 2019; Rogers et al., 2019). As a result, N accumulation is expected to increase in recent deposits. Anthropogenic nutrient loading and other wetland alterations will further impact the magnitude of N cycling and storage. In the dredged western portion of the canal, the N storage was 67% lower and the N accumulation rates 64% lower than the clogged eastern portion in recent decades. The site differences in N storage and accumulation rates between the western and eastern ends of the canal likely reflect the increased tidal flushing in the Martin Peña West in recent times, which may have lowered particulate deposition and N storage. Generally, mangrove systems that are more flushed conserve less N (Twilley and Day, 1999). In the eastern clogged end of the Caño Martin Peña, the build-up of mangrove litterfall and allochthonous organic inputs (e.g., raw sewage and domestic wastewater) may directly or indirectly contribute to high N storage and accretion rates. Nutrient over-enrichment may promote cyanobacteria and algal build-up in the Martin Peña East, which subsequently would contribute to elevated accretion and N accumulation rates in recent decades. The elevated N accumulation rates in Cubatao Forest and Sepetiba Bay (Brazil) were similarly attributed to nutrient inputs and human impacts associated with rapid urbanization (Sanders et al., 2014; Perez et al., 2020).

It may be argued that in mature, isolated mangrove systems, nutrient recycling may be of greater significance than allochthonous inputs (Twilley and Day, 1999; Alongi et al., 2004; Marchand et al., 2006). Some of the highest measures of N storage and accumulation (this study) as well as C storage were observed in the poorly flushed, least urbanized, Piñones Forest (Wigand et al., 2021). Elevated porewater NH_4_+ concentrations (mean: 1,057 ± 209 μM in the wet season; 451 ± 76 μM in the dry season), low pH (4.7; Martin et al., 2020), and low denitrification enzyme activity (Oczkowski et al., 2020b) were observed in mangrove soils at Piñones, likely due to high decomposition rates and recycling of carbon and N in the mature system (Marchand et al., 2006).

Historic hydrological alterations contributed to the isolation of the Piñones lagoon and thus its high rates of N accumulation and storage. Prior to the 1950’s the Piñones lagoon likely received water (including agricultural runoff) through a series of drainage and navigation canals from the Río Grande de Loíza and experienced periodic flushing during storms (Webb and Gómez-Gómez, 1998; Cordero, 2015). Following the damming of the Río Grande de Loíza in 1953 (Quiñones et al., 1989), flushing of the Piñones lagoon was greatly diminished, promoting a more isolated mangrove system relying primarily on nutrient recycling for growth and development. Recent N storage and accumulation rates in the Piñones Forest were 242 and 170% greater, respectively, than historical values, which may have been driven by the conservation of N in the peat in recent decades. In addition, cores from Piñones had elevated sediment accretion rates in recent decades (site mean: 5.5 mm y^−1^) compared to historic decades (site mean: 3.8 mm y^−1^) and the global median rate (2.8 mm y^−1^; 95% confidence interval 1.9–3.9) (Wigand et al., 2021). The effect of reduced flushing on N accumulation may have been augmented by expansive and dense plant matter in the mature Piñones Forest that could efficiently trap sediment particles and plant materials.

Although most of the SJBE mangrove soils were reported to contain mangrove root and leaf sources of organic matter, the Piñones mangrove soils may have been supplemented by particulate organic matter from C-4 plants (Wigand et al., 2021). The Piñones mangrove soils were characterized by atypically low *C:N* ratios (Supplementary Table 2) and an enriched δ^13^C (Wigand et al., 2021). Overland flow of C-4 plant particulates derived from surrounding agricultural lands (e.g., sugarcane), carbon recycling, and elevated porewater salinities may have contributed to the shift to a more enriched δ^13^C at Piñones (Wigand et al., 2021).

The δ^15^N values in the Martin Peña East peat were unexpectedly low, generally lower than the δ^15^N in the lagoon mangrove peat. We expected a higher δ^15^N associated with Martin Peña East due to the high inputs of sewage and human wastewater. However, as reported for the associated subtidal habitats of Martin Peña East, N fixation, possibly associated with common bluegreen algae blooms observed in Martin Peña East, may have caused a shift in the δ^15^N profile (Oczkowski et al., 2020b). Compared with atmospheric deposition and N fixation (δ^15^N of +2‰ to +8‰), and commercial, inorganic fertilizers (δ^15^N of −3‰ to +3‰), N derived from human wastewater (δ^15^N of +10‰ to +22‰) is relatively enriched in ^15^N (Kreitler et al., 1978; Gormly and Spalding, 1979; Aravena et al., 1993; McClelland et al., 1997).

Porewater *N:P* molar ratios at the SJBE mangrove sites were often much less than the typical ~19:1 ratio reported for porewater *N:P* ratios in the low intertidal zone of mangrove systems (Alongi, 1996). SJBE mangrove forest porewater *N:P* ratios generally suggested N limitation, with the notable exception of Piñones Lagoon. In Martin Peña East, PO_4_+ concentrations were very high (>45 μM) and either double (wet season) or nearly equal to (dry season) DIN concentrations. While porewater DIN was not absent (Table 1), the porewater *N:P* ratios were close to 0, and far from typical mangrove porewater *N:P* ratios (Alongi, 1996). Given the ponded nature of this stretch of the SJBE and the presumably large urban inputs from the densely settled, unsewered communities adjacent to the site, the relatively low DIN concentration and low porewater *N:P* ratios are consistent with our hypothesis of high denitrification and N fixation rates in the Martin Peña East (Lenton and Watson, 2000; Oczkowski et al., 2020a,b). The La Torrecilla Lagoon and San José Lagoon also had low porewater *N:P* ratios (≤ 10) indicative of N-limitation. Piñones had the highest measured porewater DIN concentrations (driven by high NH_4_+) of all the sites, resulting in high *N:P* ratios. In contrast, in examination of the stoichiometry of dissolved N and P in riverine water draining urbanized and more pristine watersheds in Puerto Rico, McDowell et al.(2019) reported little change in the *N:P* ratios among rivers, although the urbanized river had significant increases in the magnitude of the N and P fluxes.

Porewater salinity was more than twice that of the other locations and pH was low in mangrove soils at Piñones (Table 1). While water outflow is low in Martin Peña East, the region receives substantial amounts of inflow, via urban runoff, as evidenced by documented problems with flooding (Oczkowski et al., 2020a). In contrast, changes in tidal and riverine exchange due to man-induced alterations of the landscape (e.g., dam construction and channelization) minimizes water flow into the Piñones lagoon, except during severe storms. Hydrological alterations may have accelerated natural processes associated with mangrove development, such as more closed mineral cycles and reliance on litterfall and detritus for nutrient regeneration (Lugo and Snedaker, 1974).

The western part of the Caño Martin Peña was dredged during the 1980s as part of a waterway transportation project, which allows a greater influence of tidal waters from the adjacent San Juan Bay (Branoff, 2020a). Greater exchange with San Juan Bay waters may explain the less enriched δ^15^N and δ^34^S in the mangrove sediments at Martin Peña West compared with the clogged Martin Peña East. The lower δ^15^N in the flushed Martin Peña West may be due to diluted contributions of allochthonous Caño Martin Peña wastewater N, high water column N-fixation, and/or contributions of marine-sourced N with San Juan Bay tidal inputs. The subtropical and tropical, oligotrophic North Atlantic waters are characterized by isotopically depleted suspended particles often resulting from N-fixation by planktonic diazotrophs (Montoya et al., 2002).

Within the context of the SJBE, with its history of hydrologic modifications (e.g., canalization and dredging; Figure 1) and high porewater salinities, sediment S isotope values generally reflected a combination of two environmental factors: (1) sulfate reduction which contributes to organic matter degradation under anoxic conditions (Canfield, 2001); and (2) the degree of tidal flushing (Oczkowski et al., 2020b). High kinetic fractionation associated with sulfate reduction results in lighter sulfides and enriched sulfates, because ^32^S is more quickly reduced than ^34^S (Brownlow, 1996). Under well flushed tidal conditions, the SJBE sediment is an “open system,” where sulfate from the bay or ocean is continuously replenished, allowing isotopic fractionation resulting in low δ^34^S as reduced S. In contrast, under poorly flushed or “closed conditions” and in the case of some SJBE sites (i.e., Martin Peña East and Piñones), there is a reduced influx of marine-derived sulfate. High sulfate reduction rates under these closed conditions may cause sulfate reducers to take up more of the less energetically favorable ^34^S, causing reduced S species to retain a high δ^34^S (Oczkowski et al., 2020b). Recent genetic sequencing of lagoonal waters at Piñones revealed high representation of sulfur-oxidizing and sulfur-reducing bacteria, which are likely responsible for maintaining S homeostasis in the mostly closed system and prevent the build-up of toxic S compounds in the mangroves (Aviles and Kyndt, 2021).

In the SJBE mangroves we found that δ^34^S was depleted at depth at most sites, likely reflecting the reduced conditions found at depth in the sediment profile. In addition to reduced conditions contributing to depleted δ^34^S at depth, a possible source of enriched δ^34^S in surface sediments at Martin Peña East and San José Lagoon could be sewage discharging into the systems (Oczkowski et al., 2020a,b). Sewage sludge and discharged particles in temperate areas have δ^34^S ranging from about 0–6h (Tucker et al., 1999). The only site that had lower δ^34^S near the surface than at depth was Martin Peña West, where dredging activities increased tidal flushing with the San Juan Bay (Branoff, 2020a). Generally, we observed a trend toward lower δ^34^S in surface sediments that received greater flushing (La Torrecilla Lagoon and Martin Peña West) than more closed mangrove systems in the SJBE, supporting the hypothesis that tidal flushing is a factor that controls S isotope composition in mangrove systems, similar to δ^34^S profiles observed in the SJBE subtidal habitats (Oczkowski et al., 2020b).

Local human pressures on alteration of hydrology, tidal exchange, and soil fertility appeared to cause elevated N accumulation rates in some SJBE mangrove systems (e.g., Martin Peña East and La Torrecilla Lagoon). Anthropogenic stressors (e.g., dredging, filling, and canalization) interacted with natural drivers (e.g., tidal regime and rainfall) that add complexity to predicting nutrient cycling and storage patterns. We measured high mangrove N storage and accumulation in recent decades in Martin Peña East with the clogged canal, in La Torrecilla Lagoon with the dredged inlet, and in Piñones with the least disturbed and expansive forest. Tidal exchange through the dredged inlet at the La Torrecilla Lagoon likely provided a source of particulates and nutrients, which fueled mangrove productivity, N storage, and N accumulation. However, the dredging activities at Torrecilla Lagoon may have also caused the high spatial variability in N storage and accumulation among cores. Recycling and conservation of nutrients in the mature and hydrologically isolated Piñones Forest provided ample N for high mangrove production and N accumulation rates. The highest N accumulation rates (27.9 g ^−2^ y^−1^) in the SJBE in recent decades were observed in the clogged and eutrophied Caño Martin Peña. This is similar to elevated N accumulation rates in highly eutrophic mangrove systems in southeastern Brazil, apparently due to sewage and wastewater discharges resulting from urbanization since the 1900s (Sanders et al., 2014; Perez et al., 2020). In contrast, atmospheric N inputs from wet and dry deposition were estimated at 0.39 g ^−2^ y^−1^ in modern times and 0.15 g ^−2^ y^−1^ in pre-industrial times for the Caribbean Islands (Howarth et al., 1996). Measured wet N deposition in the Luquillo Experimental Forest (1999–2003) was 0.27 g ^−2^ y^−1^ (Ortiz-Zayas et al., 2006). These estimates suggest that wet and dry atmospheric deposition were generally not a dominant N source to the measured N accumulation rates in the SJBE mangroves.

## CONCLUSION

N accumulation rates in the SJBE mangrove soils ranged from 3.7 to 27.9 g ^−2^ y^−1^ in recent decades. Mangrove forests accumulate N from natural and anthropogenic watershed sources, tidal inputs, and N fixation. Local anthropogenic stressors may alter N storage and accumulation rates in peri-urban mangrove systems either directly by increasing N soil fertility (e.g., raw sewage and domestic wastewater inputs) or indirectly by altering geomorphology (dredging, filling, and canalization). Other larger-scale external drivers such as rainfall, wind, temperature, and storms may also affect N accumulation in the estuary. All mangrove sites in the urban SJBE, except Martin Peña West, which was dredged in the 1980s, had greater soil %N in recent decades than in historic ones.

The SJBE watershed-wide N accumulation rates in mangrove soils in recent times (0.18 Mg ha^−1^ y^−1^) were 2-times higher than the rate of food N exported as wastewater (0.08 Mg ha^−1^ y^−1^), suggesting the potential for mangroves to provide sequestration of human-derived N and act as a coastal kidney. Conservation and effective management of mangrove forests in the Anthropocene are important to the future sustainability of tropical, coastal communities throughout the world because of the many benefits provided: water quality benefits, flood protection, carbon sequestration, as well as food and fiber for millions of people (Valiela et al., 2001; Duke et al., 2007; Lugo et al., 2014; Lovelock, 2020).

## Supplementary Material

Supplement1

Supplement2

## Figures and Tables

**TABLE 1 |:** Porewater salinity and nutrient concentrations and soil pH (mean ± standard error) at mangrove sites in the wet (June 2016) and dry (February 2017) seasons in the San Juan Bay Estuary.

	Porewater					
Site/season	Salinity (PSU)	PO_4_^3−^ (μM ± se)	NH_4_^+^ (μM ± se)	DIN (μM ± se)	Soil pH	Molar *N*:*P* ratio

**MPW**						
Wet	16.0 ± 1.8	1.7 ± 0.5	24.5 ± 4.9	24.8 ± 4.9	7.1 ± 0.2	14.6
Dry	9.7 ± 1.9	3.8 ± 0.4	36.2 ± 9.0	42.0 ± 9.8	nd	11.1
**MPE**						
Wet Dry	24.7 ± 0.6	49.8 ± 8.4	20.8 ± 1.9	23.1 ± 1.9	8.2 ± 0.1	0.5
Dry	20.4 ± 1.3	93.4 ± 14.5	125.8 ± 31.4	126.8 ± 31.8	nd	1.4
**SJ**						
Wet	15.0 ± 1.0	4.2 ± 2.6	34.6 ± 13.0	35.0 ± 13.1	7.9 ± 0.1	10
Dry	17.6 ± 1.7	4.7 ± 2.3	27.8 ± 5.6	29.2 ± 5.4	nd	6.2
**Torr**						
Wet	22.7 ± 0.9	8.3 ± 2.5	24.9 ± 3.6	26.1 ± 3.5	7.6 ± 0.1	3.1
Dry	27.5 ± 0.6	9.1 ± 1.8	22.5 ± 2.6	23.1 ± 2.5	nd	2.5
**Pin**						
Wet	67.2 ± 2.0	3.9 ± 0.6	1057.3 ± 209.1	1057.3 ± 209.1	4.7 ± 0.2	271.1
Dry	72.6 ± 0.6	31.6 ± 5.5	451.0 ± 76.3	451.3 ± 76.2	nd	14.3

Salinity and pH reported in Martin et al. (2020); nd, no data. Mangrove study sites included: Martin Peña West (MPW); Martin Peña East (MPE); San José Lagoon (SJ); La Torrecilla Lagoon (Torr); Piñones Forest (Pin).

**TABLE 2 |:** Total nitrogen storage and accumulation rates for the mangroves of the San Juan Bay Estuary.

Period	AG storage	Soil storage	AG accumulation	Soil accumulation

Recent 1970–2017 ∼0–18 cm	830.7 ± 18.9 Mg	16340.0 ± 130.6 Mg	52.73 ± 1.5 Mg/y	405.3 ± 3.5 Mg/y
Historic 1930–1970 ∼19–28 cm	–	5422.5 ± 42.8 Mg	–	174.0 ± 1.7 Mg/y
Recent 1970–2017 ∼18 cm	0.36 ± 0.008 Mg/ha	7.1 ± 0.05 Mg/ha	0.02 ± 0.01 Mg/ha y	0.18 ± 0.002 Mg/ha y
Historic 1930–1970 ∼19–28 cm	–	2.3 ± 0.01 Mg/ha	–	0.08 ± 0.001 Mg/ha y

Total values (Mg and Mg y^−1^) are sums and standard errors of 100 iterations of estuary-wide interpolations, with areal values (Mg ha^−1^ and Mg ha^−1^ y^−1^) calculated by dividing the total by the mangrove area (2,313 ha). Aboveground (AG) values are calculated from weighted means of species-specific biomass nitrogen concentrations and the proportion of biomass represented by each species at each site.

**TABLE 3 |:** Nitrogen (N) inputs as food to the San Juan Bay Estuary watershed (area: 41,572 ha) and its fate.

				N in municipal solid waste		N in Wastewater	
Location	Population (# people)	N from food (Mg)	Food areal N (Mg ha^−1^ y^−1^)	N to landfill (Mg)	N recycled in compost (Mg)	N to coastal waters (Mg)	N to ground water (Mg)

Watershed	769,000	5,390	0.129	1,924	122	2371	970
MPW	3,953	27.7	0.353	9.9	0.6	17.2	
MPE	4,303	30.2	0.384	10.8	0.6	18.7	
SJ	1,729	12.1	0.154	4.3	0.3	7.5	
Torr	517	1.1	0.014	1.3	0.1	2.2	
Pin	36	0.3	0.004	0.1	0	0.2	

Mangrove site population reported for the 500 m buffer surrounding the mangrove system. Calculation details found in Supplementary Table 1.

## Data Availability

The datasets presented in this study can be found in online repositories. The names of the repository/repositories and accession number(s) can be found in the article/**Supplementary Material**.
