# Supplementary material for: Recent Nitrogen Storage and Accumulation Rates in Mangrove Soils Exceed Historic Rates in the Urbanized San Juan Bay Estuary (Puerto Rico, United States)
Source: Front For Glob Change. Author manuscript; Available in PMC 2022 Nov 12. (PMC8765364; doi:10.3389/ffgc.2021.765896)
Supplement: Supplement1 [file NIHMS1764654-supplement-Supplement1.docx]

**Supplementary Table 2.** Historic and Recent %C, %N, and δ15N (‰) means (with lower and upper bounds) and the Molar C:N Ratios of Mangrove Soils. Lower and upper bounds were the 2.5 and 97.5 percentiles of bootstrap runs (combined runs for sites with more than one core). Within time period statistical differences were based on whether boot-strapped bounds (2.5th and 97.5th) overlapped. Sites sharing the same letter within a time period are not significantly different from each other. Significance categories only reflect site/core comparisons, and not the historic vs. recent comparisons.

MPW

11.94

(10.26 - 13.13)

b

9.90

(9.06 - 10.74)

c

0.455

(0.411 - 0.484)

b

0.494

(0.443 - 0.547)

c

30.6

23.4

MPE

8.16

(6.90 - 9.03)

c

12.50

(10.83 - 14.54)

b

0.414

(0.370 - 0.450)

b

0.740

(0.607 - 0.908)

b

23.0

19.7

SJ

31.23

(27.36 - 34.58)

a

29.59

(26.20 - 32.34)

a

0.969

(0.900 - 1.020)

a

1.243

(1.142 - 1.348)

a

37.6

27.8

Torr

9.43

(4.47 - 16.33)

bcd

17.64

(12.42 - 22.20)

b

0.426

(0.236 - 0.641)

b

0.763

(0.551 - 0.989)

b

25.8

27.0

Pin

4.66

(3.68 - 5.73)

d

5.97

(5.57 - 6.5)

d

0.414

(0.295 - 0.487)

b

0.568

(0.531 - 0.609)

bc

13.1

12.3

MPW

3.88

(3.83 - 3.92)

c

2.81

(2.56 - 3.07)

c

MPE

3.85

(3.57 - 4.15)

c

3.91

(3.67 - 4.15)

b

SJ

7.24

(6.74 - 7.67)

a

5.08

(4.53 - 5.79)

a

Torr

5.55

(5.17 - 5.74)

b

5.64

(5.52 - 5.82)

a

Pin

3.75

(3.50 - 3.97)

c

3.69

(3.45 - 3.86)

b

**Site**

**δ15N**

**Historic sig. Recent sig.**

**Site**

**%C**

**Historic sig. Recent sig.**

**%N**

**Historic sig. Recent sig.**

**Molar C:N**

**Historic Recent**
